# Cervical Cancer Screening in Uganda: Determinants of Past Screening and Post-Education Uptake; A Cross-Sectional Survey

**DOI:** 10.1177/10732748261427062

**Published:** 2026-03-03

**Authors:** Ali Ssetaala, Ibrahim Muwonge, Dorcus Namulwa, Nasimu Kyakuwa, Mathias Wambuzi, Gertrude Nanyonjo, Brenda Okech, Elien De Paepe, Heleen Vermandere, Olivier Degomme

**Affiliations:** 1 Community Studies Department, UVRI-CRP Initiative Limited, Entebbe, Uganda; 2 444535 International Centre for Reproductive Health, Ghent University, Ghent, Belgium

**Keywords:** cervical cancer, screening, Uganda

## Abstract

**Introduction:**

Cervical cancer (CC) is the leading cause of cancer-related deaths among women in Uganda, largely due to late diagnosis. CC screening (CCS) is key to preventing these deaths. Awareness of past CCS, its determinants, and uptake of CCS after receiving information is crucial to informing prevention programming.

**Methods:**

A two-phase community based cross-sectional survey was conducted among 600 randomly selected women aged 25-65 years, from two Ugandan districts (Mukono and Wakiso). Participants completed a baseline questionnaire that assessed their knowledge, attitudes, and past practices related to CCS. Information on cervical cancer and screening was provided during and after the survey to encourage uptake, which was assessed three months later. Logistic regression identified factors associated with past CCS and follow-up uptake after information giving.

**Results:**

Few women [5.3%, (32/600)] were aware that Human Papillomavirus (HPV) infection causes CC. Past CCS was low [22.3%, (134/600)], associated with the age group 36-65 years (AOR= 1.9, 95% CI 1.2 - 3.2), owning a mobile telephone (AOR = 2.3, 95% CI 1.4 - 3.9), residing in a household headed by someone with tertiary or higher education (AOR=2.6, 95% CI 1.3 - 5.0), self-reported HIV infection (AOR=10.5, 95% CI 5.2 - 21.3), awareness of the location of CCS services (AOR=3.2, 95% CI 1.3 - 7.8), and awareness that accessing the CCS location was not expensive (AOR=2.3, 95% CI 1.3 - 4.0).

The uptake of CCS following information provision was 40.3%, (236/585), associated with employment (AOR=2.7, 95% CI 1.2 - 5.8), moderate-high income (AOR=1.6, 95% CI 1.0 - 2.6), and prior receipt of CCS services (AOR 6.7, 95% CI 4.0 -11.2).

**Conclusion:**

CCS remains low but is higher among women with better socioeconomic status, awareness of services, and HIV infection. Targeted strategies addressing awareness and motivating women to get screened can boost screening uptake.

## Background

Cervical cancer (CC) remains the leading cause of cancer-related morbidity and mortality in Uganda.^1^ CC contributes about 7,000 new cases and over 4,000 deaths annually, accounting for nearly 20% of all cancer cases and deaths nationally and more than one-third of all female cancers.^1,2^ Alarmingly, the majority of cases are diagnosed at advanced stages, drastically reducing survival prospects and underscoring systemic failures in prevention, early detection, and timely treatment.^3,4^ This late-stage presentation persists despite the availability of cost-effective, evidence-based interventions such as HPV vaccination, screening, and early treatment of precancerous lesions.

Cervical cancer screening (CCS) is delivered through a nationally organized provider-initiated program often integrated into reproductive health and HIV care services, led by the Ministry of Health in close collaboration with the Uganda Cancer Institute (UCI) and international partners. Implementation is primarily within public health facilities, at national, regional, and district levels, complemented by periodic outreach campaigns across districts. Screening is mainly among women aged 25 - 49 years, annually for HIV-positive and triennially for HIV-negative individuals. Screening may also be offered to women outside this range depending on risk factors. Visual Inspection with Acetic Acid (VIA) is the most widely used method; HPV DNA testing is also available at a few sites. Pap smear (cytology) and colposcopy are used in higher-level facilities (e.g., UCI, national referrals, and some private hospitals). In public and partner-supported health facilities, screening services are free, with no fees for VIA, HPV testing, or Pap smears funded through the National Cancer Management and Capacity Building Project in Uganda (CANCAP-UG project), UCI, and development partners [Korea Foundation for International Healthcare (KOFIH), The U.S. President’s Emergency Plan for AIDS Relief (PEPFAR)]. Private providers may charge fees and offer free or subsidized screening during campaigns.

Existing literature from Uganda identifies several interrelated factors that contribute to low cervical cancer screening (CCS) uptake. These include limited knowledge and awareness of cervical cancer and available services,^5-7^ low perceived risk, fear of diagnosis, and pervasive myths and misconceptions about screening procedures.^3^ Women’s educational attainment consistently emerges as a significant predictor of screening behavior, with higher education associated with greater health literacy and service utilization.^3,8^ Social support or its absence from spouses, family, and peers further mediates women’s health-seeking decisions, particularly in patriarchal contexts where male approval is often required for care.^3,8^ Additionally, health system constraints, including geographic and financial inaccessibility, shortages of trained personnel, inconsistent service availability, and negative provider attitudes, further impede access.^3,9^

Despite this literature, critical gaps remain. Most Ugandan studies on CCS have been facility-based or conducted in one district, limiting generalizability. Few have employed population-based designs to simultaneously assess coverage, self-reported screening behavior, and multilevel determinants in regions bearing the highest disease burden, like the central region.^10^ Moreover, there is limited integration of these research findings into actionable frameworks that can inform the national scale-up of the World Health Organization’s (WHO) aligned elimination strategies.

Uganda is a signatory to the WHO global strategy to eliminate cervical cancer as a public health problem by 2030, which sets three key targets: 90% of girls fully vaccinated against HPV by age 15; 70% of women screened with a high-performance HPV test by ages 35 and 45; and 90% of women identified with precancerous or invasive lesions receiving timely treatment.^11^ However, national cervical cancer screening coverage remains below 40%,^5^ and HPV vaccination coverage, though improving, still falls short of the 90% target.^12,13^ These gaps reflect a complex web of structural, economic, sociocultural, and health system barriers that have been documented in prior Ugandan studies but not yet comprehensively analyzed in high-burden regions, such as central Uganda.

This study addresses these gaps by providing community-level evidence from central Uganda, a region with high cervical cancer incidence but with disproportionate related literature.^10^ We examined the magnitude of CCS coverage and associated factors, screening services knowledge, and the impact of information provision on screening services uptake among women living in Mukono and Wakiso districts, Uganda.

This study is part of the Community-based HPV Screening Implementation in Low-Income Countries” (CHILI Project),^14^ contributing to cervical cancer screening research in resource-limited settings. Our findings inform policy and practice by identifying modifiable barriers that, if addressed through targeted interventions (e.g., community health education, male engagement, task-shifting, and integration of screening into primary care), could accelerate progress toward the WHO 90-70-90 elimination targets.

## Theoretical Framework

The Theory of Planned Behavior (TPB) and the associated Theory of Reasoned Action (TRA) provide a framework for understanding cervical cancer screening behavior by exploring the relationships among behavior, beliefs, attitudes, and intentions.^15^ Both theories postulate that human actions are guided by intentions, which are in turn shaped by a person’s attitude towards undertaking the cervical cancer screening behavior, beliefs about whether individuals who are important to the person approve or disapprove of the behavior (subjective norms or perceived social pressure), and, under the TPB, perceived behavioral control (PBC), which reflects perceived ease or difficulty in performing the behavior.^15,16^ PBC reflects a person’s perception of the ease or difficulty of performing the behavior, which is influenced by their control beliefs about the presence of factors that may facilitate or impede performance. TPB argues that when individuals perceive that they have high control over a behavior, their intentions are more likely to translate into actual action. TRA speculates that the most immediate predictor of a person’s voluntary behavior is their intention to perform that behavior. The stronger an individual’s intention, the more likely they are to participate in a behavior. The intention is, in turn, determined by: (1) the individual’s attitude toward the behavior, which is their overall positive or negative evaluation of performing it; and (2) subjective norms, which refer to the perceived social pressure to perform or not perform the behavior, based on the beliefs about what important others think they should do.^17^

## Methods

### Study Design and Setting

**Figure 1.**
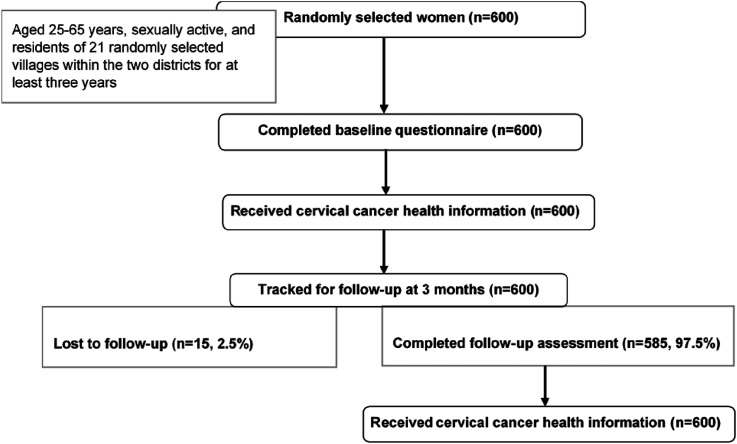
Study flow diagram

This cross-sectional survey estimated CCS coverage and associated factors, screening services knowledge and preferences, and the impact of information provision on screening services uptake among women living in two Ugandan districts. The study involved 600 randomly selected women aged 25-65 years, who were sexually active, and residents from 21 randomly selected villages within the two districts for at least three years (see Figure 1).

The study was conducted in two neighboring districts, Wakiso and Mukono, both located in central Uganda and surrounding the capital city, Kampala. These districts were purposively selected to capture a mix of urban, peri-urban, and rural settings, thereby enhancing the generalizability of findings across diverse socioeconomic and healthcare access contexts within the region. Wakiso is one of Uganda’s most populous and rapidly urbanizing districts, with relatively better access to health facilities, including both public and private providers offering cervical cancer screening (primarily via visual inspection with acetic acid, VIA). Mukono, while experiencing urban growth, retains a larger rural population and faces more pronounced constraints in healthcare infrastructure.

Despite these contextual differences, the two districts share key demographic, cultural, and health system characteristics: they belong to the same administrative region (central Uganda), serve populations predominantly from the Baganda ethnic group, operate under similar district-local government health policies, and are served by comparable tiers of public health facilities (Health Centers II–IV and district hospitals). Moreover, both districts have been included in national and donor-supported cervical cancer prevention programs (e.g., those led by the Uganda Ministry of Health with support from the Cervical Cancer Prevention Program, CANCAP-UG project, UCI, and the Program for Appropriate Technology in Health, PATH), resulting in comparable exposure to public health messaging and CCS availability. Given these similarities in sociocultural context, health system structure, and cervical cancer control programming and in the absence of evidence suggesting fundamentally different screening behaviors or knowledge patterns between the two districts, we treated them as a single epidemiological unit for analytical purposes. A preliminary bivariate analysis confirmed no statistically significant differences in key outcome variables (past cervical cancer screening and post-education uptake) between districts (p = 0.75 and p = 0.83, respectively). Therefore, data from Wakiso and Mukono were pooled to increase statistical power and provide a more robust estimate of determinants associated with cervical cancer screening behavior in this representative central Ugandan population. This approach enhances the external validity of our findings for similar settings across central Uganda while maintaining internal validity through consistent data collection protocols and standardized educational interventions across both sites.

According to the Uganda Bureau of Statistics (UBOS), the Mukono district’s 2023 projected population was approximately 789,700 people, while that of Wakiso district was 3.7 million people.^18^

### Sample Size Calculation

The sample size (600) was calculated using Cochran’s formula for sample size with a finite population correction (FPC).^19^ FPC was applied to the formula: n = N*X/(X + N – 1). where, X = [(Zα/2)^2^ *P*(1-P)]/e^2^. Zα/2 (1.96) was the critical value of the normal distribution at α/2 (e.g., for a confidence level of 95%, α was 0.05, and the critical value was 1.96). P was the sample proportion (21%),^20^ e is the margin of error (0.05), and N was the population size (females 25-65 years, 511,291 for Wakiso and 140,361 for Mukono). Expecting 21% coverage,^20^ with a precision of 5% and 95% confidence interval, the required sample size in each study district was 255. This number was raised to 300 (600 from both districts) to cater for a 15% none response rate. Stratified random sampling was used to select sub-counties from each of the eleven counties in the two districts. Ten sub-counties were selected from Mukono district and eleven from Wakiso district. Study villages were then randomly selected from the selected sub-counties. Ten villages were randomly selected from each of the selected Mukono district sub-counties, recruiting 30 women from each of the villages. Eleven villages were randomly selected from the selected Wakiso district sub-counties, recruiting 27 women from each of the villages (except for three villages where 28 women were recruited from each). Study participants were randomly selected from each village based on the household register at the Local Council (LC)1 office, with one participant being selected per household.

Approval of community leaders was sought before inviting women to participate. The research team approached the randomly selected women in every household, provided detailed information, and requested them to participate. If a selected woman was unavailable, a follow-up visit was scheduled. Eligible and willing participants received an information sheet in English or Luganda, read aloud if needed. For illiterate participants, this was done in the presence of an impartial witness. Those who understood and agreed to participate signed an informed consent form and received a copy.

Trained research assistants administered a baseline face-to-face quantitative survey on household characteristics, socio-economic status, participants’ socio-demographic characteristics, cervical cancer knowledge, attitudes, and practices. The data collection instrument was a structured questionnaire developed for the CHILI Survey and Discrete Choice Experiment (DCE) in Uganda to assess knowledge, attitudes, practices, and socio-economic factors related to cervical cancer prevention and screening. It was designed using REDCap,^21^ and adapted to the local context through expert review, translation, and back-translation, and community (through the community advisory board) input to ensure cultural relevance. The tool underwent content validation and was pilot tested in a selected Ugandan community (Nakiwogo) to refine clarity, flow, and response options. Quality control measures, including standardized procedures and reviewer checks, were incorporated to enhance reliability and accuracy.

The survey included cervical cancer information provision as part of an intervention to improve CCS uptake. The cervical cancer information was in a leaflet/flyer format, delivered by trained research assistants after piloting. Standardization was maintained by training the research assistants on delivery, i.e., face-to-face, one-on-one, being read out to the study participant over 15 minutes in either English or the local language. The message contained the following information;

1. What is cervical cancer?
  Cervical Cancer develops in a woman’s cervix, which is the entrance to the uterus from the vagina. Almost all (99%) cervical cancer cases are linked to infection with high-risk human papillomavirus (HPV), an extremely common virus transmitted through sexual contact. Cervical Cancer is the leading cause of female cancer in Uganda and the most common cancer in women aged 15 to 44 years.
2. Risk factors. A woman is at risk of developing cervical cancer if;
  i. She has multiple sexual partners without a condom
  ii. Having sex before the age of 18 years
  iii. Smoking
  iv. Weakened immune system
  v. Chemical exposure
3. Symptoms of cervical cancer;
  i. Abnormal vaginal bleeding
  ii. Abnormal vaginal discharge
  iii. Pelvic pain
  iv. Heavier longer-lasting period
4. Unusual bleeding may occur after;
  i. After sexual intercourse
  ii. After the menstrual period
  iii. After menopause
  iv. After pelvic examination
5. Treatment;
  i. Surgery may include total removal of the uterus
  ii. Radiation treatment
  iii. Using anticancer medications
6. Have a PAP Smear or VIA test;
  A pap smear is a simple quick vaginal examination to check if your cervix is healthy. A special instrument, the speculum is used to hold the vaginal opening for the health worker to see the cervix. Some cells are gently wiped off the cervix and sent to the laboratory for testing. The test results are then shared with you.
7. Why should a woman go for a PAP Smear or VIA test?
  Pap smears help detect abnormal cells that may later become the cancer of the cervix. Testing is done every 3 years and every year if you are HIV infected.
8. Your health right;
  Dignity, respect, and privacy when being attended to. Health worker to explain procedures to you. Informed consent.
9. Your responsibilities;
  i. Responsibility for your own health.
  ii. Go back for the pap smear results.
  iii. Go back for any follow if advised to.
  iv. Give the correct contact or location details.
10. Prevention is better than cure. Cervical cancer is preventable, by getting vaccinated with the HPV vaccine.
11. Cervical cancer is treatable if detected early.
12. Study site contact information.

This was conducted during January- December 2024 within the women’s homes or workplaces, or any other convenient location of their choice, where confidentiality would be maintained.

A follow-up survey was administered three months later, via telephone, assessing the self reported uptake of CCS at follow-up, after information provision. The follow-up was premised on the assumption that the data from the baseline survey would be dissimilar from the follow-up survey in some aspects (CCS uptake after information provision, especially among women with prior experience). Data were collected on computer tablets and managed using REDCap electronic data capture tools hosted at Ghent University.^21,22^

### Statistical Analysis

This analysis aimed at answering the following questions;

1. What is the CCS coverage, and what factors are associated with having ever been screened?
2. What awareness is related to cervical cancer screening?
3. What is the uptake of CCS at follow-up, and what factors are associated with this uptake?

#### Study Variables

##### Outcome (Dependent) Variables

Ever been screened for CC (Yes/No), and CCS uptake after the information provision (Yes/No). These are strong predictors of future screening and are consistently included in CC services studies.^23-28^

##### Independent Variables

• *Participants’ characteristics*: Participants’ age (years), highest education level, religious affiliation, marital status, main occupation (in line with the common demographic characteristics used in Ugandan national health surveys), self-reported HIV infection status (HIV is a well-established biological cofactor for cervical carcinogenesis), and possession of a mobile phone (this may be associated with health services usage^29-31^).
• *Household’s characteristics*: Household head gender, Household head’s highest education level, household head’s main occupation, religious affiliation, marital status, household residence (urban, peri-urban, rural) [demographic characteristics used in national household surveys], household economic status (moderate-high if any household member owns a motorcycle, car, boat, agricultural land, or private health insurance).
• *Knowledge and awareness*: Ever heard of CC (Yes/No), ever heard of CCS, awareness of CCS location, awareness of HPV causing CC. These variables measure specific etiological knowledge, a key component of the Health Belief Model, and are widely used in CCS studies.^23,32,33^
• *Reasons for lack of screening*: Difficult to find time for screening, expensive getting to the CCS clinic, getting CCS is up to the individual, difficult to find transport for CCS, years since the last CCS, and possession of a mobile phone.
Awareness of the location of CCS services (yes/no), and perception that CCS is not expensive (Yes, Neutral, No) reflect Andersen’s Behavioral Model of health services use, where awareness of service location and cost are enabling resources that facilitate service utilization.^32^
• *Willingness for CCS after information provision* (Yes/No). Educational information may influence the uptake of CCS services.^24-27,34^

Frequency tabulation of women’s characteristics, household’s characteristics, knowledge, and awareness related to CC, reasons for lack of CCS, and willingness for CCS after information was obtained from ever screening for CC and CCS uptake was made to understand related factors. Bivariate chi-square tests were used to assess the associations between the independent variables above and the outcomes (ever screening for CC and CCS uptake) at a 95% significance level.

To understand the factors associated with ever being screened for CC and CCS uptake, logistic regression models were fitted at multivariable analysis. The independent variables mentioned above were included in the initial multivariable models based on previous literature, biological plausibility, or a statistical significance (P-value ≤ 0.2) at bivariable analysis. Residence, religion, number of lifetime pregnancies, and number of rooms in the house were excluded from the initial multivariable predictors of ever receiving CCS model as they had a P-value > 0.2 at bivariable analysis.^35-37^ Residence, marital status, education, household head gender, household head education, and willingness to have CCS after the information session were excluded from the multivariable model for predictors of CCS uptake after the information session because they had a P-value > 0.2 at bivariable analysis. The most suitable models were selected based on having the lowest Akaike’s information criterion (AIC) and Bayesian information criterion (BIC) values. We removed variables that did not improve the models (education, religion, marital status, main occupation, household head gender, head of household education, household economic status, participant ever heard of CC, participant ever heard of CCS, awareness that HPV causes CC, and getting CCS being up to the individual) or those that were highly correlated (ever heard of CC, household head main occupation, household head education) with other variables (ever heard of CCS, main occupation, education) in the models, with the final predictors in the model having the lowest P-values, model AIC, and BIC values. Adjusted coefficients, P-values, and 95% confidence intervals (CI) were used to report associations. All analyses were done using STATA^®^ version 17.^38^ The STROBE cross-sectional reporting guidelines were used to guide the manuscript writing.^39^

### Ethical Considerations

The study was approved by the Uganda Virus Research Institute Research Ethics Committee (UVRI-REC) on 19^th^ September 2023 [Approval registration number GC/127/973] and the Uganda National Council of Science and Technology on 13^th^ November 2023 [Approval registration number HS3200ES].

All women were enrolled after providing written, voluntary informed consent. All study procedures were conducted in accordance with the Helsinki Declaration of 1975, as revised in 2024. All study participants’ details have been de-identified.

## Results

### Participants’ Characteristics

Participants’ median (Inter Quartile Range) age was 39 (31-48) years. Women resided in rural [58% (348/600)], peri-urban [28.2% (169/600)], and urban [13.8% (83/600)] areas. More than half of the participants were married [57.8%, (347/600)], with fewer women having formally studied up to tertiary level education [8.2%, (49/600)]. Most women were in non-farming occupations [69.5%, (417/600)], and owned a mobile telephone [69.2%, (415/600)]. See Table 1.

**Table 1.** Participant sociodemographic and household characteristics in relation to ever screening (n=600)

	Ever screened		Overall	COR	95% CI
	Yes, N (%)	No, N (%)	(N=600)		
Participant characteristics	134 (22.3)	466 (77.7)			
Median (IQR) age (years)		39 (31-48)		1.0	1.0 - 1.1
Age (Completed years)			**P<0.05**		
25-35	36 (26.9)	195 (41.9)	231 (38.5)	(Ref)	
36-65	98 (73.1)	271 (58.1)	369 (61.5)	2.0	1.3 – 3.0
Education			**P=0.11**		
Other	20 (14.9)	56 (12.0)	76 (12.7)	(Ref)	
Primary & Secondary	98 (73.1)	377 (80.9)	475 (79.2)	0.7	0.4 - 1.3
Tertiary	16 (11.9)	33 (7.1)	49 (8.2)	1.4	0.6 - 3.0
Religion			**P=0.65**		
Other	29 (21.6)	119 (25.5)	148 (24.7)	(Ref)	
Christian	73 (54.5)	240 (51.5)	313 (52.2)	1.2	0.8 - 2.0
Muslim	32 (23.9)	107 (23.0)	139 (23.2)	1.2	0.7 - 2.2
Marital status			**P=0.20**		
Married	71 (53.0)	276 (59.2)	347 (57.8)	(Ref)	
Not married	63 (47.0)	190 (40.8)	253 (42.2)	0.6	0.3 - 1.4
Main occupation			**P<0.05**		
Farming	31 (23.1)	152 (32.6)	183 (30.5)	(Ref)	
Non-Farming	103 (76.9)	314 (67.4)	417 (69.5)	1.6	1.0 - 2.5
HIV infection status^β^			**P<0.05**		
Not infected	92 (70.2)	436 (96.3)	528 (90.4)	(Ref)	
Infected	39 (29.8)	17 (3.8)	56 (9.6)	10.4	5.0 - 21.3
Possession of a mobile phone			**P=0.18**		
No	35 (26.1)	150 (32.2)	185 (30.8)	(Ref)	
Yes	99 (73.9)	316 (67.8)	415 (69.2)	1.3	0.9 - 2.1
Lifetime pregnancies			**P=0.38**		
Up to five	79 (59.0)	294 (63.1)	373 (62.2)	(Ref)	
Over five	55 (41.0)	172 (36.9)	227 (37.8)	1.2	0.8 - 1.8
Household characteristics					
Household head - gender			**P=0.18**		
Male	78 (58.2)	301 (64.6)	379 (63.2)	(Ref)	
Female	56 (41.8)	165 (35.4)	221 (36.8)	1.3	0.9 - 1.9
Household head - education			**P<0.05**		
Non-tertiary	115 (85.8)	428 (91.8)	543 (90.5)	(Ref)	
Tertiary	19 (14.2)	38 (8.2)	57 (9.5)	1.9	1.0 - 3.4
Household head - main occupation			**P=0.09**		
Farming	31 (23.1)	143 (30.7)	174 (29.0)	(Ref)	
Non-Farming	103 (76.9)	323 (69.3)	426 (71.0)	1.5	0.9 - 2.3
Residence			**P=0.52**		
Rural	72 (53.7)	276 (59.2)	348 (58.0)	(Ref)	
Peri-urban	42 (31.3)	127 (27.3)	169 (28.2)	1.3	0.8 - 2.0
Urban	20 (14.9)	63 (13.5)	83 (13.8)	1.2	0.7 - 2.1
Economic status			**P=0.17**		
Low Income	57 (42.5)	168 (36.0)	225 (37.5)	(Ref)	
Moderate-high Income	77 (57.5)	298 (64.0)	375 (62.5)	0.8	0.5 - 1.1
Number of rooms in the house			**P=0.27**		
One	47 (35.1)	188 (40.3)	235 (39.2)	(Ref)	
Over one	87 (64.9)	278 (59.7)	365 (60.8)	1.3	0.8 - 1.9

### Cervical Cancer Awareness

The majority of women had ever heard of CC [94.7%, (568/600)], though very few [5.3%, (32/600)] knew that Human Papillomavirus (HPV) is the cause of cervical cancer. The majority [89%, (507/600)] were aware of the existence of CC screening and where to locate the services [87%, (441/600)]. However, over half felt it was difficult accessing CCS services [54.7%, (328/600)], and expensive getting the services [53.2%, (319/600)]. See Table 2.

**Table 2.** Participant knowledge and awareness characteristics in relation to ever screening (n=600)

	Ever screened		Overall	COR	95% CI
	Yes, N (%)	No, N (%)	(N=600)		
Knowledge and awareness	134 (22.3)	466 (77.7)			
Ever heard of CC			**P<0.05**		
No	2 (1.5)	30 (6.4)	32 (5.3)	(Ref)	
Yes	132 (98.5)	436 (93.6)	568 (94.7)	4.5	1.1 - 19.3
Ever heard of CCS			**P<0.05**		
No	0	63 (14.4)	63 (11.0)	(Ref)	
Yes	132 (100)	375 (85.6)	507 (89.0)	1	
Awareness of the CCS location			**P<0.05**		
No	7 (5.3)	59 (15.7)	66 (13.0)	(Ref)	
Yes	125 (94.7)	316 (84.3)	441 (87.0)	3.3	1.5 - 7.5
Awareness of HPV causing CC			**P<0.05**		
Don’t know	121 (90.3)	447 (95.9)	568 (94.7)	(Ref)	
Yes	13 (9.7)	19 (4.1)	32 (5.3)	2.5	1.2 - 5.3
Reasons for lack of screening					
Difficulty finding time for CCS			**P=0.08**		
Yes	46 (34.3)	199 (42.7)	245 (40.8)	(Ref)	
No	88 (65.7)	267 (57.3)	355 (59.2)	1.4	1.0 - 2.1
Expensive getting to the CCS clinic			**P<0.05**		
Yes	69 (51.5)	250 (53.7)	319 (53.2)	(Ref)	
Neutral	8 (6.0)	83 (17.8)	91 (15.2)	0.3	0.2 - 0.8
No	57 (42.5)	133 (28.5)	190 (31.6)	1.6	1.0 - 2.3
Getting CCS is up to the individual			**P=0.14**		
No	1 (0.7)	15 (3.2)	16 (2.7)	(Ref)	
Yes	133 (99.3)	451 (96.8)	584 (97.3)	4.4	0.6 - 33.8
Difficult transport for CCS			**P=0.15**		
Agree	76 (56.7)	226 (48.5)	302 (50.3)	(Ref)	
Neutral	3 (2.2)	23 (4.9)	26 (4.3)	0.4	0.1 - 1.3
Disagree	55 (41.0)	217 (46.6)	272 (45.3)	0.8	0.5 - 1.1

### Cervical Cancer Screening

**Figure 2.**
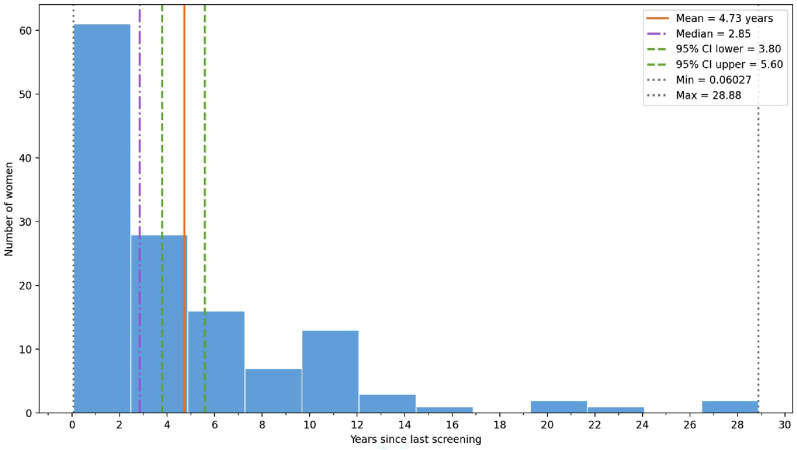
Temporal distribution of years since last cervical cancer screening (n=134)

Less than one in four women had ever been screened for cervical cancer in their lifetime [22.3%, (134/600)], with screening times ranging from 1 to 6 (mean of 1.3 times, 95% CI 1.2-1.4), and time since last CC screening ranging from 0.06 years (22 days) to 28.9 years (mean of 4.7 years, 95% CI 3.8 - 5.6). See Figure 2.

### Factors Associated With Ever Being Screened for CC

Ever been screened reporting was higher among HIV infected women [69.6%, (39/56)] compared to HIV negative women [17.4%, (92/528)]. Over half of HIV uninfected women [52.7%, (48/91)] had received their recent CCS service more than three years ago, while nearly 7 in 10 HIV infected women [69.2%, (27/39)] had received the most recent CCS more than one year ago. Self-reported HIV infection was associated with ever having been screened for cervical cancer (AOR=10.5, 95% CI 5.2 - 21.3).

Older age, more than 35 years (age group 36-65 years) was associated with ever been screened for CC (AOR= 1.9, 95% CI 1.2 - 3.2), as well as women staying in households where the household head had attained tertiary level education (AOR=2.6, 95% CI 1.3 - 5.0), and those who owned a mobile telephone (AOR = 2.3, 95% CI 1.4 - 3.9). Awareness of CCS location was also associated with ever being screened for cervical cancer (AOR=3.2, 95% CI 1.3 - 7.8). Women who thought that it was not too expensive to reach the health facility for CCS were more likely to have ever received screening (AOR = 2.3, 95% CI 1.3 - 4.0). Transport to the health facility, being perceived as not difficult, was associated with ever been screened for cervical cancer (AOR = 0.5, 95% CI 0.3 - 0.9). See Table 3.

**Table 3.** First and final adjusted logistic regression models of factors associated with ever being screened for CC

Characteristics	First multivariable model		Final multi-variable model	
	AOR	95% CI	AOR	95% CI
Participant characteristics				
Age group (Completed years)				
25-35	(Ref)		(Ref)	
36 & Older	1.9	1.1- 3.2	1.9*	1.2 - 3.2
Marital status				
Not married	(Ref)			
Married	0.6	0.3 - 1.4		
Occupation				
Farming	(Ref)			
Non-Farming	1.2	0.7 - 2.1		
HIV infection				
Negative	(Ref)		(Ref)	
Infected	10.4	5.0 - 21.3	10.5*	5.2 - 21.3
Possession of a mobile phone				
No	(Ref)		(Ref)	
Yes	2.1	1.2 - 3.5	2.3*	1.4 - 3.9
Household characteristics				
Household head - gender				
Male	(Ref)			
Female	0.8	0.3 - 1.7		
Household head - education				
Non-tertiary	(Ref)			
Tertiary	2.5	1.2 - 5.0	2.6*	1.3 - 5.0
Economic status				
Low	(Ref)	(Ref)		
Moderate- High	0.9	0.5 - 1.4		
Knowledge and awareness				
Awareness of the CCS location				
No	(Ref)	(Ref)		
Yes	3.1	1.3 - 7.6	3.2*	1.3 - 7.8
Awareness of HPV causing CC				
No	(Ref)			
Yes	1.5	0.6 - 3.7		
Reasons for lack of screening				
Difficult to find time for CCS				
Agree	(Ref)		(Ref)	
Disagree	1.4	0.9 - 2.3	1.4*	0.9 - 2.4
Expensive getting to the CCS clinic				
Yes	(Ref)		(Ref)	
Neutral	0.4	0.2 - 1.1	0.4	0.2 - 1.1
Disagree	2.2	1.2 - 3.9	2.3*	1.3 - 4.0
Getting CCS is up to the individual				
No	(Ref)			
Yes	2.1	0.2 - 18.8		
Difficult transport for CCS				
Agree	(Ref)		(Ref)	
Neutral	1.0	0.2 - 4.4	0.9	0.2 - 3.7
Disagree	0.5	0.3 - 0.9	0.5*	0.3 - 0.9

*Statistically significant, P-value ≤ 0.05

### Uptake of Cervical Cancer screening after the information provision and associated factors

Nearly all women (93.5%) were willing to be screened for CC shortly following an information session on CC. 585 women [97.5%, (585/600)] were followed up via a telephone call (see Figure 1), of which two of every five women [40.3%, (236/585)] had taken up CC screening at follow-up. More than half of the women [56.8% (134/236)] who took up CC screening at follow-up had never received prior CCS. See Table 4.

**Table 4.** Factors Associated With Uptake of CCS After Information Provision (n=585)

Characteristic	CCS uptake		Overall	COR	95% CI	AOR	95% CI
	No, N (%)	Yes, N (%)	585				
Participant characteristics	349 (59.7)	236 (40.3)					
Willingness for CCS after information			**P=0.65**				
Unwilling	24 (6.9)	14 (5.9)	38 (6.5)	(Ref)			
Willing	325 (93.1)	222 (94.1)	547 (93.5)	1.2	0.6 - 2.3		
Age (Years)			**P≤0.05**				
25-35	145 (41.6)	79 (33.5)	224 (38.3)	(Ref)			
36-65	204 (58.4)	157 (66.5)	361 (61.7)	1.4	1.0 - 2.0	1.4	0.9 - 2.2
Education			**P=0.72**				
Other	56 (12.0)	20 (14.9)	76 (12.7)	(Ref)			
Primary & Secondary	377(80.9)	98 (73.1)	475 (79.2)	0.9	0.5 - 1.4		
Tertiary	33 (7.1)	16 (11.9)	49 (8.2)	0.7	0.4 - 1.6		
Religious affiliation			**P= 0.06**				
Other	278 (79.7)	172 (72.9)	450 (76.9)	(Ref)		(Ref)	
Muslim	71 (20.3)	64 (27.1)	135 (23.1)	1.5	1.0 - 2.2	1.4	0.9 - 2.3
Marital status			**P=0.46**				
Not married	143 (41.0)	104 (44.1)	247 (42.2)	(Ref)			
Married	206 (59.0)	132 (55.9)	338 (57.8)	0.9	0.6 - 1.2		
Employment status			**P<0.05**				
Unemployed	43 (12.3)	14 (5.9)	57 (9.7)	(Ref)		(Ref)	
Employed	306 (87.7)	222 (94.1)	528 (90.3)	2.2	1.2 - 4.2	2.7*	1.2 - 5.8
Main occupation			**P=0.15**				
Farming	114 (23.1)	64 (32.6)	178 (30.4)	(Ref)		(Ref)	
Non-Farming	235 (67.3)	172 (72.9)	407 (69.6)	1.3	0.9 - 1.9	1.5	1.0 - 2.5
Lifetime pregnancies			**P=0.12**				
Up to five	225 (64.5)	137 (58.0)	362 (61.9)	(Ref)			
Over five	124 (35.5)	99 (42.0)	223 (38.1)	1.3	0.9 - 1.8		
HIV infection status^є^			**P<0.05**				
Not infected	325 (94.8)	194 (84.0)	519 (90.4)	(Ref)		(Ref)	
Infected	18 (5.2)	37 (16.0)	55 (9.6)	3.4	1.9 - 6.2	1.3	0.6 - 2.8
Household characteristics							
House head gender			**P=0.80**				
Male	221 (63.3)	147 (62.3)	368 (62.9)	(Ref)			
Female	128 (36.7)	89 (37.7)	217 (37.1)	1.0	0.7 - 1.5		
Household head education status			**P=0.89**				
Other	81 (23.2)	51 (21.6)	132 (22.6)	(Ref)			
Primary & Secondary	236 (67.6)	162 (68.6)	398 (68.0)	1.1	0.7 - 1.6		
Tertiary	32 (9.2)	23 (9.8)	55 (9.4)	1.1	0.6 - 2.2		
Number of rooms in the house			**P<0.05**				
One	149 (42.7)	79 (33.5)	228 (39.0)	(Ref)			
More than one	200 (57.3)	157 (66.5)	357 (61.0)	1.5	1.1 - 2.1		
Household head’s main occupation			**P=0.11**				
Farming	110 (31.5)	60 (25.4)	170 (29.1)	(Ref)			
Non-Farming	239 (68.5)	176 (74.6)	415 (70.9)	1.4	0.9 - 2.0		
Residence			**P=0.78**				
Rural	206 (59.0)	133 (56.4)	339 (58.0)	(Ref)			
Peri-urban	97 (27.8)	68 (28.8)	165 (28.2)	1.1	0.7 - 1.6		
Urban	46 (13.2)	35 (14.8)	81 (13.8)	1.2	0.7 - 1.9		
Economic status			**P=0.09**				
Low Income	141 (40.4)	79 (33.5)	220 (37.6)	(Ref)		(Ref)	
Moderate-high Income	208 (59.6)	157 (66.5)	365 (62.4)	1.4	1.0 - 1.9	1.6*	1.0 - 2.6
Knowledge and awareness							
Ever heard of CC			**P<0.05**				
No	24 (6.9)	6 (2.5)	30 (5.1)	(Ref)			
Yes	325 (93.1)	230 (97.5)	555 (94.9)	2.8	1.1 - 7.0		
Ever heard of CCS			**P=0.16**				
No	39 (11.9)	19 (8.3)	58 (10.4)	(Ref)			
Yes	288 (88.1)	211 (91.7)	499 (89.6)	1.5	0.8 - 2.7		
Awareness of the CCS location			**P<0.05**				
Unaware	46 (16.0)	20 (9.5)	66 (13.2)	(Ref)			
Aware	242 (84.0)	191 (90.5)	433 (86.8)	1.8	1.0 - 3.2	1.2	0.6 - 2.1
Getting CSS is up to the individual			**P<0.05**				
No	14 (4.0)	2 (0.9)	16 (2.7)	(Ref)		(Ref)	
Yes	335 (96.0)	234 (99.1)	569 (97.3)	4.9	1.1 - 21.7	2.8	0.5 - 15.1
Ever screened for CC			**P<0.05**				
Never screened	319 (91.4)	134 (56.8)	453 (77.4)	(Ref)		(Ref)	
Ever screened	30 (8.6)	102 (43.2)	132 (22.6)	8.1	5.1 - 12.7	6.7*	4.0 - 11.2

*Statistically significant, *p≤0.05*. ^є^11 women missing HIV infection status.

Employed women were thrice as likely to take up CCS after information provision relative to unemployed women (AOR=2.7, 95% CI 1.2 - 5.8). Women employed in non-farming occupations (AOR=1.5, 95% CI 1.0 - 2.5) and those with moderate to high-income status (AOR=1.6, 95% CI 1.0 - 2.6) were more likely to take up CCS after information provision. Having been previously screened for cervical cancer was associated with a 7 times higher likelihood of taking up cervical cancer screening after information provision (AOR 7.0, 95% CI 4.0 - 11.2). See Table 4.

## Discussion

This cross-sectional survey was conducted to establish the current coverage, knowledge about cervical cancer, and uptake of CCS after information provision among two central Ugandan districts.

The proportion of women ever been screened for CC in their lifetime was far below the WHO global strategy ‘90-70-90’ target for elimination of CC by 2030. This low proportion of women ever having been screened for cervical cancer, though similar to some Ugandan trends,^8,11,40^ is much higher than the estimated overall national screening coverage.^41^ The low coverage of CCS underscores the urgent need to enhance screening efforts among women to improve their health and help the country stay on track to meet WHO targets. The low CCS coverage could be due to limited awareness of cervical cancer screening, as previously documented by Isabirye and colleagues.^40^ Our work indicated that nearly all women were aware of CC, with the majority of women who had never been screened having heard about CC.

Awareness of CCS services location was associated with ever having been screened for cervical cancer, although we could not determine whether awareness of location occurred before or after receipt of CCS. This is similar to previous work done in Eastern Uganda and sub-Saharan Africa, indicating the importance of knowledge of CCS location on the uptake of screening services.^7,23,42^ Awareness of the CCS location may increase knowledge of screening, which is associated with the uptake of cervical cancer services and improvement of health-seeking behaviors among at-risk women.^23,42^

Awareness that accessing the CCS location or clinic was not expensive was related to having been screened for CC. Awareness of access to the CCS location not being expensive may be part of CC related information and knowledge that has been previously related to the uptake of CCS.^23,42^ Awareness that accessing the CCS location is not expensive might imply that if access to the CCS location is subsidized or made free of charge through public-private partnerships or universal health coverage, it might increase uptake of screening services by women, as indicated by previous literature.^42^

The observed associations align well with the TPB and TRA theoretical framework. Awareness that CCS is not expensive and knowledge of where to access CCS services likely contribute to a more positive attitude toward screening. When individuals perceive screening as affordable and accessible, they are more inclined to view it favorably, increasing intention and actual uptake. Costs related to CCS are one of the barriers to screening in Uganda.^3^ When women believe the service is affordable, they are more likely to evaluate the CCS behavior positively, as it removes the significant financial burden negative outcome. This belief directly contributes to a favorable attitude, which maps directly into the PBC of the TPB. When women know where to go for CCS and that it’s affordable, they feel more capable of taking up CCS.

Having been screened for CC was associated with older age, probably because CC screening information targets older women who are at increased risk, with screening being linked to a reduction of CC burden and deaths among older age groups.^43^ Older women tend to have co-morbidities that require frequent seeking of health care services, where they end up getting CC information and screening. Increases in CCS uptake have been linked to older age in SSA.^23^

Household head tertiary education was related to having ever been screened for CC, probably because highly educated partners are likely to encourage and support their women and daughters in seeking health care, including CCS.^23,42^ This is also in line with the TPB and TRA theories,^15^ residing in a household headed by someone with tertiary or higher education may reflect exposure to social norms that value preventive healthcare. A household head with higher education is more likely to be aware of the importance of preventive health measures, including CCS, and to communicate this value to other household members. This reinforces perceived social approval (subjective norms). In Uganda’s mainly collectivist cultures, such household-level influences might strongly shape individual intentions. Education is also often linked to more access to health-related information, awareness of cervical cancer, and better health-seeking behavior, including CCS.^42,44^ Contrary to previous similar work,^3,8,45-47^we did not find a statistically significant association between participants’ education and CCS, probably because most women in the central region depend on their partners for health seeking. Partner education status has previously been linked to movement for reproductive health services.^48^

Ownership of a mobile phone was related to having ever been screened for CC. Phone ownership has been linked to socio-economic status, increased access to cervical cancer information, and uptake of screening services.^29-31^ Women with cell phones are more likely to access cervical cancer information sources and to be part of social networks that improve health awareness and increase uptake of CCS.^34^ Mobile phone ownership may increase personal connections with social media, health workers, relatives, and peers who have undergone prior screening, and may eventually become sources of cervical cancer information, which has been linked to increased screening. This finding adds to the growing evidence of the role of mobile phones in improving cervical cancer awareness, prevention, and screening among women in Uganda.^29,49^ The finding aligns well with the PBC of the TPB: the enhanced contact with health information, appointment reminders, and teleconsultations through mobile phone ownership increases perceived control over accessing screening.

Self-reported HIV infection was strongly related to having ever been screened for CC. In Uganda, women living with HIV are often enrolled in routine care that includes CCS as part of comprehensive HIV management. This institutionalized pathway increases both PBC (screening is embedded in care) and subjective norms (healthcare providers strongly recommend it), aligning with the TPB predictions. The PEPFAR program has been supporting access to free CCS services for HIV infected women attending public hospitals and some health center level IV facilities for four years.^50^ This provided increased access to screening services among HIV infected women and might have prompted these women, who could have perceived themselves to be at increased risk of CC, to take up the free service. Despite many HIV infected women taking up CCS, the majority had had their recent screening over a year ago, indicating the need for more information on CCS, even among the majority of HIV infected women previously screened, as the screening guidelines are different for HIV infected women.^51^

Information giving improved uptake of CCS among women, findings similar to previous work elsewhere.^26,27,52^ Information provision could have helped women understand issues related to accessing CCS services, including the location of the service. Information provision facilitates access to accurate guidance, alleviates screening hesitancy, misconceptions, and empowers women to make informed decisions towards CCS. Information giving provides details on why women should get screened and facilitates individual risk self-assessment by providing details on the risk factors and symptoms of CC disease. We observed that uptake of CCS following information provision was associated with being employed, working non-farming occupations, moderate to high income status, and previous CCS. This observation is central to TRA/TPB. Information campaigns aim to change the underlying beliefs that form attitudes, subjective norms, and PBC. However, the theory predicts that such information will only lead to behavior change if it successfully shifts these core constructs. The fact that uptake after information was linked to employment, income, and prior screening suggests that the information alone was insufficient for those with low PBC. For them, the information may have improved their attitude towards CCS or intention to screen, but without the structural capacity (income, time, experience) to act, the intention did not translate into CCS behavior. In contrast, women with higher PBC (due to income, employment, or past experience) were able to act on their newly formed or strengthened intentions to take up CCS.

Employed women, those with moderate-to-high income status, and women with prior CCS experience were related to uptake of screening after information provision. These associations expand on the TPB and TRA theoretical framework. Employment and moderate-to-high income improve structural capacity (time, transport, financial resources) to seek CCS services, strengthening PBC. Women with prior experience of screening (past uptake) may hold more favorable attitudes after the information session due to health system familiarity and reduced fear or uncertainty. Women who had prior CCS experience might have known what to expect and feel more confident repeating the behavior, in line with PBC.

According to the TPB and TRA, intention is the immediate precursor to behavior. In this study context, women with favorable attitudes (e.g., due to awareness of affordability to reach a health facility), supportive subjective norms (e.g., from educated household heads), and high PBC (e.g., due to mobile phone ownership, moderate-to-high income, or past CCS experience) are more likely to form strong intentions to screen and subsequently do so, especially when prompted by information provision.

Programs for improving screening among women in these districts should consider having cervical cancer information sessions as part of interventions to improve uptake.

A limitation of this study is its cross-sectional design, which precludes the establishment of temporality, that is, the ability to determine whether the identified exposures (e.g., household head education, HIV status, awareness of service location, employment, income) preceded the outcomes (past screening or post-information uptake). Data on these exposures and outcomes were collected at the same point in time, making it difficult to ascertain causal relationships or even the direction of associations. Therefore, while the findings offer important insights into correlates of CCS in this population, they should be interpreted as associations rather than causal relationships. Randomized controlled trials or prospective cohort studies would be better suited to establish temporality and evaluate the causal impact of specific determinants on screening behavior.

Another limitation of this study is the reliance on self-reports to assess CCS behavior. This approach introduces the potential for information bias. Participants might have inaccurately reported whether they had ever undergone CCS due to:

i. Overreporting: Social desirability bias may have led some women to report having been screened when they had not, especially if they perceived screening as a socially approved health behavior. This is particularly relevant in settings where health education campaigns have elevated the perceived importance of CCS.
ii. Lack of verification with medical records: The study did not validate self-reported CCS history against facility-based medical records. Without such verification, it is challenging to confirm the accuracy of reported CCS, including whether the correct procedure (e.g., VIA or HPV testing) was actually performed.
iii. Recall bias: Even among women who had truly undergone CCS, the accuracy of recall regarding the timing, frequency, or nature of the screening event may be compromised, especially if the last screening occurred several years prior. While the outcome of interest was “ever screened” (a binary measure), imperfect recall could still affect the consistency and reliability of responses, particularly in a population with generally low health literacy and limited familiarity with cervical cancer terminology, as suggested by the low awareness of HPV as a cause of cervical cancer (5.3%).

These limitations may have led to an overestimation of true past CCS prevalence and could influence the strength and direction of associations identified in the logistic regression models. For example, if overreporting was more common among certain subgroups (e.g., HIV infected women), this could introduce differential misclassification (HIV infected women might think harder and more accurately about their past CCS (motivated by increased risk perception and their regular engagement with health services) than women without HIV infection, who may not have a reason to recall minor details.

Despite these limitations, the use of self-report remains a practical and widely accepted method in population-based surveys, especially in resource-limited settings, such as Uganda, where centralized health records are often unavailable or incomplete. Future studies would benefit from incorporating objective verification of screening history where feasible, or employing validated survey instruments designed to minimize recall and social desirability biases.

## Conclusion

CCS coverage is still low among women in central Uganda, being associated with women’s age, household head education, and mobile phone ownership. Provision of cervical information, including information on the location and accessibility of services, is crucial to the uptake of screening services, especially among the unemployed, low-income status women who have never screened for cervical cancer in their lifetime.

## Data Availability

The dataset used and analyzed during the current study is publicly available at https://doi.org/10.6084/m9.figshare.30530963.v1 (Ref. 53).

## Appendix

### List of Abbreviations

AOR: Adjusted Odds Ratio
CANCAP-UG: National Cancer Management and Capacity Building Project in Uganda project
CC: Cervical cancer
CCS: Cervical cancer screening
CHILI: Community-based HPV Screening Implementation in Low-Income Countries project
CI: Confidence Interval
DNA: Deoxyribonucleic acid
HIV: Human Immunodeficiency Virus
HPV: Human Papilloma Virus
KOFIH: Korea Foundation for International Healthcare
PAP: Papanicolaou smear
PBC: Perceived Behavior Control
PEPFAR: President’s Emergency Plan for AIDS Relief
SSA: Sub-Saharan Africa
STROBE: Strengthening the Reporting of Observational Studies in Epidemiology
TPB: Theory of Planned Behavior
TRA: Theory of Reasoned Action
U.S: United States
VIA: Visual Inspection with Acetic Acid
WHO: World Health Organization
